# In time: misuse and overuse of amino acid formulas in cow milk allergy

**DOI:** 10.1016/j.rpped.2015.08.003

**Published:** 2015

**Authors:** Jon A. Vanderhoof

**Affiliations:** a Boston Children's Hospital, Boston, USA

Allergic proctocolitis and enterocolitis have been successfully treated with extensively hydrolyzed formulas for many years.^1^ In 1997, our group and de Boissieu et al.^2^ in Paris reported independently two series of patients with cow milk protein induced allergy that failed to respond to extensively hydrolyzed formulas, but ultimately responded therapeutically to an amino acid-based infant formula.^3^ These patients were subsequently challenged with an extensively hydrolyzed formula and indeed their symptoms recurred, confirming intolerance to the extensively hydrolyzed product. IgE binding epitopes have been demonstrated in both extensively hydrolyzed whey and casein and are thought to be responsible for these reactions.^4^^,^ 5 An amino acid-based formula was successfully utilized to treat eosinophilic esophagitis, confirming that this disorder is in fact an allergic process amenable to dietary therapy.^6^

The percentage of infants with cow milk protein allergy who do not tolerate an extensively hydrolyzed formula appears be low. Traditionally, the percentage is thought to be around 5%, but some have postulated that it may be on the rise.^7^ Despite the low incidence of intolerance to extensively hydrolyzed protein, the use of amino acid formulas has vastly exceeded the predicted usage in many countries, despite a significant increase in the cost of therapy. Commercial promotions and government reimbursement policies in some areas may have been partially responsible for this phenomenon. It is also quite true, however, that many clinicians are not aware of the literature supporting the use of extensively hydrolyzed and amino acid formulas.

Amino acid formulas are sometimes utilized by clinicians because they believe the clinical response will be more rapid or the relapse rate will be significantly lower resulting in a greater degree of patient satisfaction. This is especially true when cost to the patient is not a significant deterrent. There have been no randomized studies conducted to determine the response rate to an amino acid formula versus an extensively hydrolyzed protein formula in allergic infants. Severe enterocolitis is often thought to be an indication for initial use of an amino acid formula, and even recommended in some guidelines for the management of allergic infants.^8^ However, no studies have ever demonstrated increased efficacy of an amino acid-based formula in this situation. Amino acid-based products are often utilized in allergic esophagitis based upon the original report demonstrating efficacy in these patients. One study, however, using an extensively hydrolyzed product in adults, demonstrated a positive symptomatic response and provided an economical alternative therapy.^9^ None have been done in children.

Is there reason not to utilize amino acid formulas in every allergic baby other than cost? Recently, data suggest that some of the peptides present in extensively hydrolyzed formulas, especially those based on casein, may facilitate the induction of tolerance.^10^^,^ 11 Specific peptide fragments have now been identified that may play a role in this process, and this hypothesis has been preliminarily verified in animal studies.^11^ Earlier development of tolerance to cow milk protein of course is a much desired outcome in the treatment of allergic disease. It also appears that *Lactobacillus* GG, a well-studied probiotic organism (*Lactobacillus rhamnosus* ATC 51033), may significantly augment this process.^12^ It is possible other organisms might do this as well but further research is needed before such statements can be made with confidence. Nonetheless, the induction of tolerance is a key goal in allergy management and whatever can be done to facilitate this process is certainly important. Oral tolerance induction may also be possible through desensitization, and preliminary data look positive here.^13^

Another issue that should be addressed is the utilization of strategies to prevent or reduce the likelihood of the development of protein allergy in at risk populations. Extensively hydrolyzed casein-based formulas also play a role here, and while not equally efficacious, partially hydrolyzed whey-based formulas may also play a role. There are however conflicting data with partial hydrolysates.^14^ Interestingly, extensively hydrolyzed whey-based formulas do not appear to be effective.^15^ Finally, breast-feeding is an excellent and cost-effective way to reduce the risk of cow milk protein allergy in high-risk populations, and should be the first option if available. The probiotic *Lactobacillus* GG also appears to be helpful in the situation.^16^ Further studies are needed to determine the ideal age for introduction of proteins into the diet to prevent allergy, as some population-based studies have suggested that early introduction may be ideal.^17^

Food allergies, and particularly cow milk protein allergy, along with other allergies and autoimmune disorders, are becoming more common and more significant health care issues.^18^ The interventions discussed here and other modalities to effectively treat and prevent food allergies will become increasingly important as time progresses.
