# Supplementary material for: In time: misuse and overuse of amino acid formulas in cow milk allergy
Source: Rev Paul Pediatr. 2015 Oct-Dec;33(4):379–80. doi: 10.1016/j.rpped.2015.08.003 (PMC4685555; doi:10.1016/j.rpped.2015.08.003)
Supplement: Translation

# Translation: Em tempo: uso indevido e excessivo de fórmulas de aminoácidos na alergia ao leite de vaca

**Authors:** Jon A. Vanderhoof

**Affiliations:** a Boston Children’s Hospital, Boston, EUA

Proctocolite alérgica e enterocolite têm sido tratadas por vários anos com sucesso com fórmulas extensamente hidrolisadas.^1^ Em 1997, o nosso grupo e o grupo de Boissieu et al.^2^ de Paris relataram independentemente duas séries de pacientes com alergia induzida à proteína do leite de vaca que não responderam às fórmulas extensamente hidrolisadas, mas responderam terapeuticamente a uma formulação infantil à base de aminoácidos.^3^ Esses pacientes foram subsequentemente expostos a uma fórmula extensamente hidrolisada e, de fato, os seus sintomas retornaram, confirmando a intolerância ao produto extensivamente hidrolisado. Epítopos de ligação com IgE foram demonstrados no soro de leite e na caseína extensamente hidrolisados e são considerados os responsáveis por essas reações.^4^^,^ 5 Uma fórmula à base de aminoácidos foi usada com sucesso para tratar a esofagite eosinofílica e confirmou que essa doença é na verdade um processo alérgico passível de terapia dietética.^6^

A porcentagem de crianças com alergia à proteína do leite de vaca que não toleram uma fórmula extensamente hidrolisada parece ser baixa. Tradicionalmente, a porcentagem parece ser de aproximadamente 5%, mas alguns têm postulado que esse percentual pode estar aumentando.^7^ Apesar da baixa incidência de intolerância à proteína extensamente hidrolisada, o uso de fórmulas de aminoácidos excedeu consideravelmente o previsto em muitos países, a despeito de um aumento significativo do custo da terapia. Promoções comerciais e políticas de reembolso do governo em algumas áreas podem ter sido parcialmente responsáveis por esse fenômeno. É também bastante evidente, no entanto, que muitos médicos não têm conhecimento da literatura que apoia o uso de fórmulas extensamente hidrolisadas e de aminoácidos.

Fórmulas de aminoácidos são às vezes usadas por médicos porque eles acreditam que a resposta clínica será mais rápida ou a taxa de recaída será significativamente menor, o que resultará em um maior grau de satisfação do paciente. Isso é especialmente verdadeiro quando o custo para o paciente não é um impedimento significativo. Não há estudos randomizados feitos para determinar a taxa de resposta a uma fórmula de aminoácidos *versus* uma fórmula de proteína extensamente hidrolisada em crianças alérgicas. A enterocolite grave é muitas vezes considerada uma indicação para o uso inicial de uma fórmula de aminoácidos e até mesmo recomendada em algumas diretrizes para o tratamento de bebês alérgicos.^8^ No entanto, nenhum estudo jamais demonstrou maior eficácia de uma fórmula à base de aminoácidos nessa situação.

Produtos à base de aminoácidos são frequentemente usados em esofagite alérgica com base em um relato original que demonstra a eficácia nesses pacientes. Um estudo, no entanto, com o uso de um produto extensivamente hidrolisado em adultos demonstrou uma resposta sintomática positiva e forneceu uma opção econômica de terapia.^9^ Nenhum desses estudos foi feito em crianças.

Existe outra razão para não usar fórmulas de aminoácidos em todos os bebês alérgicos que não seja o custo? Recentemente, dados sugerem que alguns dos peptídeos presentes em fórmulas extensamente hidrolisadas, especialmente aquelas à base de caseína, podem facilitar a indução de tolerância.^10^^,^ 11 Fragmentos específicos de peptídeos já identificados podem desempenhar um papel nesse processo e essa hipótese foi preliminarmente verificada em estudos com animais.^11^ O desenvolvimento precoce da tolerância à proteína do leite de vaca é, obviamente, um desfecho muito desejado no tratamento da doença alérgica. Parece também que o *Lactobacillus* GG (LGG), um organismo probiótico bastante estudado (*Lactobacillus rhamnosus* ATC 51033), pode aumentar significativamente esse processo.^12^ É possível que outros organismos também possam ter a mesma ação, mas é necessário maior investigação antes que tais afirmações possam ser feitas com confiança. No entanto, a indução de tolerância é um objetivo-chave no tratamento da alergia e tudo o que puder ser feito para facilitar esse processo é certamente importante. A indução de tolerância oral também pode ser possível por meio de dessensibilização, como demonstrado por dados preliminares.^13^

Outra questão que deve ser abordada é o uso de estratégias para prevenir ou reduzir a probabilidade de desenvolvimento de alergia à proteína em populações de risco. Fórmulas à base de caseína extensamente hidrolisadas desempenham essa função e, embora não sejam igualmente eficazes, fórmulas à base de soro de leite parcialmente hidrolisado podem ter o mesmo papel. No entanto, existem dados conflitantes a respeito de hidrolisados parciais.^14^Curiosamente, fórmulas à base de soro de leite extensivamente hidrolisado não parecem ser efetivas.^15^ Finalmente, a amamentação é uma forma excelente e com ótimo custo-benefício para reduzir o risco de alergia à proteína do leite de vaca em populações de alto risco e deve ser a primeira opção, caso disponível. O probiótico LGG também parece ser útil nessa situação.^16^ São necessários mais estudos para determinar a idade ideal para a introdução de proteínas na dieta para evitar alergia, uma vez que alguns estudos populacionais têm sugerido que a introdução precoce pode ser o ideal.^17^

Alergias alimentares e, especialmente alergia à proteína do leite de vaca, juntamente com outras alergias e doenças autoimunes, estão se tornando os problemas de saúde mais comuns e mais significativos.^18^ As intervenções discutidas aqui e outras modalidades para tratar de forma efetiva e prevenir alergias alimentares se tornarão cada vez mais importantes com o passar do tempo.
